# 

**Published:** 1861

**Authors:** 


New Series, Vol. 4. Nos. 6 & 7. Whole Series, Vol. 18, Nos. 6 & 7.

### THE CHICAGO

### MEDICAL JOURNAL.

DANIEL BRAINARD, M. D.,
J. ADAMS ALLEN, M. D.,
Editors and Proprietors.

JUNE & JULY, 1861.

CHICAGO:

WM. PIGOTT, BOOK AND JOB PRINTER, 139 CLARK STREET.

1861.

